# Climate variations of Central Asia on orbital to millennial timescales

**DOI:** 10.1038/srep36975

**Published:** 2016-11-11

**Authors:** Hai Cheng, Christoph Spötl, Sebastian F. M. Breitenbach, Ashish Sinha, Jasper A. Wassenburg, Klaus Peter Jochum, Denis Scholz, Xianglei Li, Liang Yi, Youbing Peng, Yanbin Lv, Pingzhong Zhang, Antonina Votintseva, Vadim Loginov, Youfeng Ning, Gayatri Kathayat, R. Lawrence Edwards

**Affiliations:** 1 Institute of Global Environmental Change, Xi’an Jiaotong University, Xi’an, China; 2 Department of Earth Sciences, University of Minnesota, Minneapolis, USA; 3 Institute of Geology, Universität Innsbruck, Innsbruck, Austria; 4 Sediment- & Isotope Geology, Institute for Geology, Mineralogy & Geophysics, Ruhr-Universität, Bochum, Germany; 5 Godwin Laboratory, Department of Earth Science, University of Cambridge, Cambridge, UK; 6 Department of Earth Sciences, California State University Dominguez Hills, Carson, USA; 7 Institute for Geosciences, Johannes Gutenberg-Universität Mainz, Germany; 8 Climate Geochemistry Department, Max Planck Institute for Chemistry, Mainz, Germany; 9 Sanya Institute of Deep Sea Science and Engineering, CAS, Sanya, China; 10 School of Geological Sciences and Mineral Resources, Lanzhou University, Lanzhou, China; 11 Nuffield Department of Clinical Medicine, University of Oxford, Oxford, UK; 12 Ekaterinburg Speleological Club (ESC), Ekaterinburg, Russia

## Abstract

The extent to which climate variability in Central Asia is causally linked to large-scale changes in the Asian monsoon on varying timescales remains a longstanding question. Here we present precisely dated high-resolution speleothem oxygen-carbon isotope and trace element records of Central Asia’s hydroclimate variability from Tonnel’naya cave, Uzbekistan, and Kesang cave, western China. On orbital timescales, the supra-regional climate variance, inferred from our oxygen isotope records, exhibits a precessional rhythm, punctuated by millennial-scale abrupt climate events, suggesting a close coupling with the Asian monsoon. However, the local hydroclimatic variability at both cave sites, inferred from carbon isotope and trace element records, shows climate variations that are distinctly different from their supra-regional modes. Particularly, hydroclimatic changes in both Tonnel’naya and Kesang areas during the Holocene lag behind the supra-regional climate variability by several thousand years. These observations may reconcile the apparent out-of-phase hydroclimatic variability, inferred from the Holocene lake proxy records, between Westerly Central Asia and Monsoon Asia.

Central Asia (CA) constitutes the core area of the Asian continent spanning across the mid-latitudes from the Caspian Sea in the west to western China in the east (Supplementary Fig. 1). The fresh water availability in this arid-semiarid region is a highly crucial issue and yet, significant questions remain concerning dynamical processes that govern CA climate change on various timescales and their teleconnections with the Asian monsoon (AM). For example, a number of recent model/reanalysis results demonstrate a close coupling between the CA and AM climates and the mechanism behind^1^,^2^,^3^,^4^,^5^, while the lake proxy records suggest that the Westerly CA and the AM climate variations are nearly anti-phased during the Holocene^6^,^7^,^8^. Previously, using a single cave oxygen isotope (δ^18^O) proxy record from Kesang cave, Cheng *et al*.^9^ have shown that climate variability in eastern CA on orbital timescale is similar to the AM, consistent with the modeling results^1^,^2^,^5^,^10^. However, because Kesang cave is located in the eastern fringe of CA with rainfall season during boreal summertime, rather than during boreal wintertime as typically of most part of CA, the question remains whether the climate variability reconstructed from the Kesang record is representative of the CA, which is dominated by wintertime precipitation. In addition, it is critical to obtain other proxy data from Kesang and/or other caves in CA that can characterize the local hydroclimatic variations in cave regions, which will allow a direct comparison to the existing lake records^6^,^7^. Here, we present precisely-dated and high-resolution multi-proxy cave records from Tonnel’naya (Ton) cave, Uzbekistan, and Kesang cave, western China (Supplementary Fig. 1). These records reveal temporal patterns of both supra-regional and local climate variations over most of the last 135 and 500 ka (thousand years), respectively. We use these records to infer distinct CA climate modes, which we link to the AM variability and CA lake records on multiple timescales.

## Cave and Climate Settings

Ton and Kesang caves are located at 38°24′N, 67°14′E, 3226 m a.s.l., and 42°52′N, 81°45′E, 2000 m a.s.l., with precipitation occurring mainly during wintertime and summertime, respectively. Relative humidity and air temperature are nearly constant in Ton cave (~100% and ~3.1 °C, respectively, Supplementary Fig. 2). Kesang cave climate setting and environmental characteristics are described in detail in ref. 9. The continental climate of CA is dynamically complex. Present-day moisture transport to CA occurs mainly via the Westerlies from the Atlantic Ocean, the Mediterranean and the Caspian Seas, together with local recycling^11^,^12^. Occasionally, the moisture from the Indian monsoon region might affect the southeastern area of CA^11^. The wintertime precipitation is a prevailing feature in CA, except in its most eastern part where spring-summer rainfall dominates (Supplementary Fig. 1). This is mainly because that the Siberian High can turn the Westerly moisture transport to eastern CA southward during winter, and subsequent retreat of the Siberian High in spring gives rise to Westerly moisture transport into the region during spring-summer. The two cave locations are ideal for the purpose of this study because they represent the winter (Ton cave) and summer (Kesang cave) precipitation regimes of CA, respectively. This allows us to test whether climate variability, previously inferred from the Kesang δ^18^O record, is truly of supra-regional nature.

## Samples and Methods

**Figure 1:**
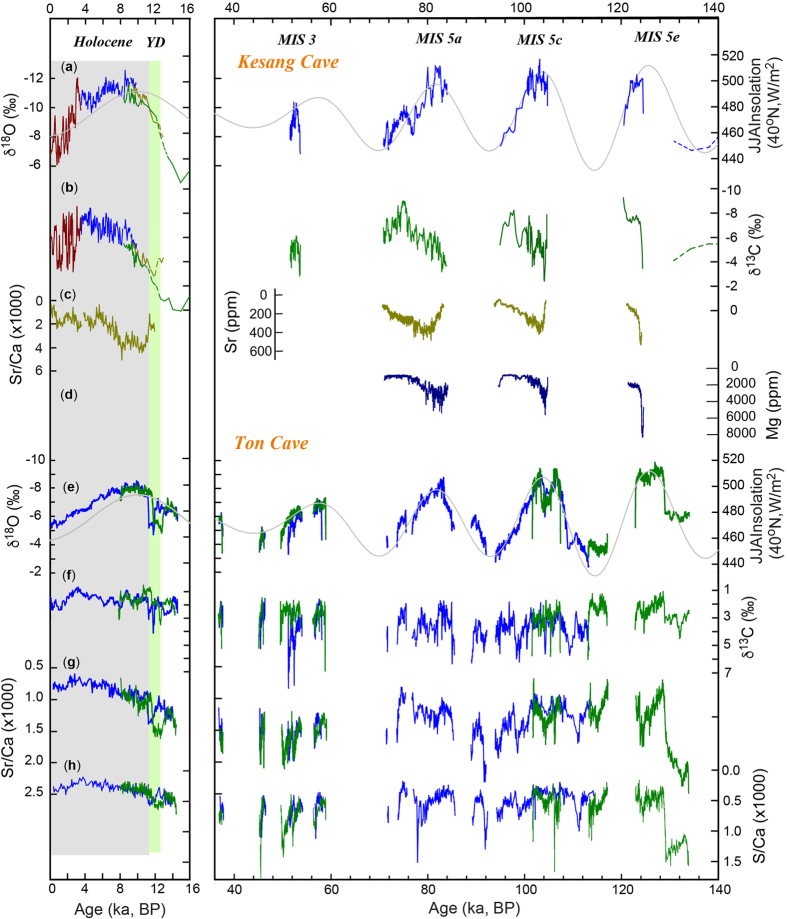
Speleothem records from Kesang and Ton caves. (**a**–**d**) Kesang δ^18^O, δ^13^C, Sr and Mg records. (**e**–**h**) Ton δ^18^O, δ^13^C, Sr/Ca and S/Ca records. BP, before present, where present is 1950. Kesang and Ton δ^18^O records are virtually the same, indicating the supra-regional climate variability. Both Kesang (**b**–**d**) and Ton (**f**–**h**) δ^13^C and trace element records are broadly similar, but different from the corresponding δ^18^O records, suggesting distinct hydroclimatic variability in cave areas. The YD event in TON-1 (green) and TON-2 (blue) records indeed exhibits some offset because of the dating uncertainty. The YD event is well dated in the TON-1 record (high growth rate) in contrast to that in TON-2 record, which has much larger age uncertainties because of very low growth rate around the event. However, within the dating uncertainties, the YD events in both records they agree with each other.

**Figure 2:**
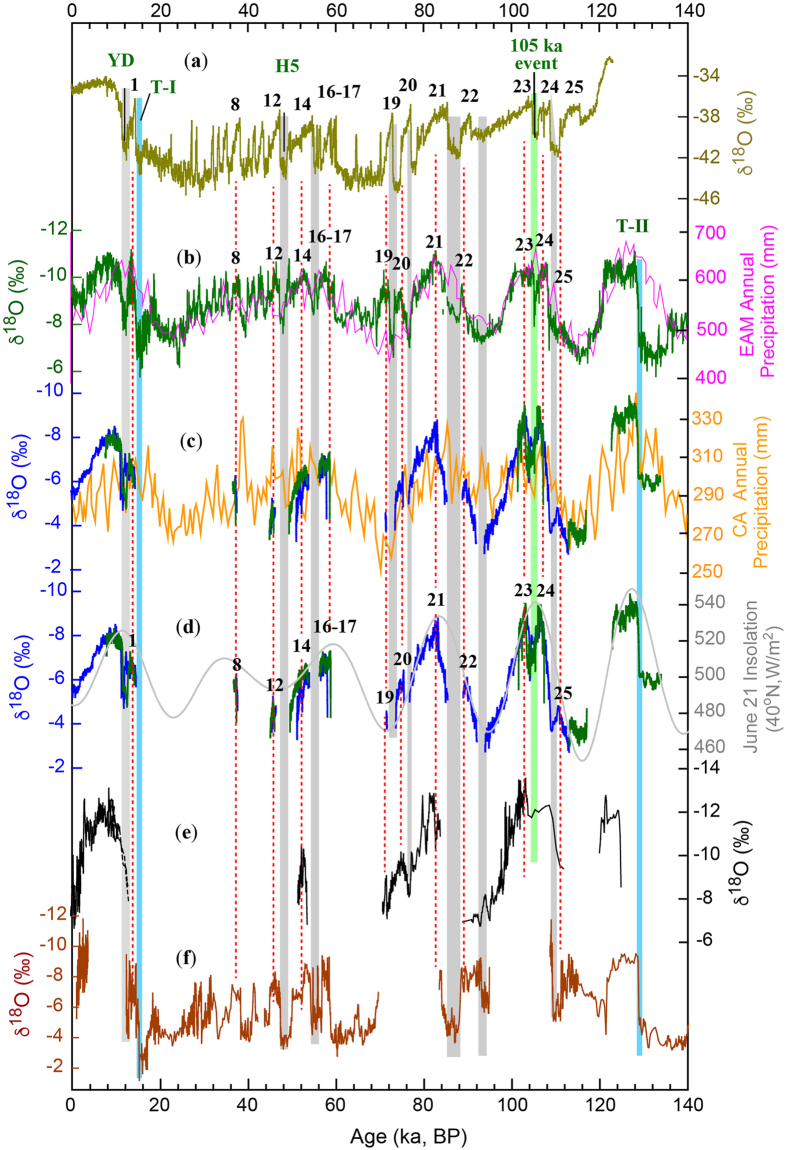
Comparison of climatic records and model results over the past 140 ka. (**a**) Greenland NGRIP ice-core δ^18^O record^57^. (**b**) East Asian monsoon δ^18^O record (green)^18^ and modeled annual precipitation (pink)^5^. (**c**) Ton δ^18^O records (TON-1 in green and TON-2 in blue) and modeled annual precipitation in CA (orange)^5^. (**d**) Ton δ^18^O records (blue and green) and June 21 insolation at 40°N^58^. (**e**) Kesang δ^18^O records^9^. (**f**) Indian monsoon record^21^. Blue bars depict glacial terminations I and II. Green bar shows the 105 ka event. Grey bars and dashed lines indicate correlations among stadial/YD/Heinrich-events and interstadials, respectively. The numbers indicate Greenland interstadials (**a**), AM interstadials (**b**) and CA interstadials (**d**).

Using a recently improved ^230^Th dating technique^13^, we obtained 72 ^230^Th dates for two stalagmite samples from Ton cave (TON-1 and TON-2, collected in 2011) (Supplementary Fig. 3) and 4 ^230^Th dates for a new stalagmite from Kesang cave (KS08-6, collected in 2008) (Supplementary Tables S1–S3) in Xi’an Jiaotong University, China. All ages are in stratigraphic order within dating uncertainties. Linear interpolations between ^230^Th dates are used to establish chronologies (Supplementary Fig. 4). δ^18^O and δ^13^C values were measured on ~6280 subsamples from TON-1 and TON-2 at the Universität Innsbruck, Austria, and 46 for KS08-6 at Xi’an Jiaotong University (Supplementary Tables S1 and S3). High-resolution trace element data were obtained using a Thermo Finnigan Element 2 Inductively Coupled Plasma Mass Spectrometer (ICPMS) coupled to a New Wave UP213 Laser Ablation System at the Max Planck Institute for Chemistry, Mainz, Germany (Kesang cave sample KS08-2) (Supplementary Table S4), and using the micro-X-ray fluorescence (XRF) scan (the remaining samples in this study) at the School of Earth Sciences, China University of Geosciences, China (see Methods). Extensive Hendy^14^ (Supplementary Fig. 5) and replication tests^15^,^16^ between contemporaneous portions of stalagmite TON-1 and TON-2 records (Figs 1 and 2) suggest minimal kinetic disequilibrium influence on isotopic values.

## Results and Cave Paleoclimate Proxies

New Ton cave records presented in this study include δ^18^O, δ^13^C and trace element data, covering the most of the last 135 ka with a temporal-resolution of ~40 years or better. Kesang cave records include new δ^13^C and trace element data, incorporated with previous published δ^18^O records^9^, covering the most of the last 500 ka, with a temporal-resolution of ~350 years or better. Both Ton and Kesang records are precisely dated with sufficient temporal-resolution to characterize orbital to millennial scale climate variability—one that we focus here. Overall, the Ton and Kesang cave δ^18^O records are similar, characterized by large-amplitude (~5–6‰) variations, whereas the temporal pattern of δ^13^C and trace element variations in the two cave records are generally different from each other (Fig. 1). The climatic interpretation of these cave proxies are explained below.

### Cave δ^18^O proxy

Following the same line of reasoning used by Cheng *et al*.^9^ in explaining Kesang δ^18^O records, we interpret the Ton δ^18^O variability, particularly the large amplitude changes occurring on orbital-millennial scales, in terms of changes in the δ^18^O value of precipitation (δ^18^O_p_). We reason that temperature change alone cannot explain the large range in Ton δ^18^O values (~6‰) (Fig. 1) because this would require unreasonably large changes in cave temperature (up to 24 °C^17^).

A number of studies suggests that on orbital to millennial timescales, δ^18^O_p_ variations in tropical and subtropical latitudes are primarily controlled by large-scale changes in atmospheric circulation accompanied by changes in rainfall amount, temperature, seasonality, as well as moisture source and trajectories^9^,^16^,^18^. The δ^18^O_p_ variability, in principle, results from the spatially-integrated, upstream precipitation processes between moisture source and cave site, and processes at the local scale, such as amount effect at each cave site^18^,^19^,^20^,^21^. Most modeling studies support this notion^5^,^22^,^23^,^24^. Indeed, a high-degree of similarity observed among δ^18^O_p_ records from distant caves in a same climate system thus, indicates a common climate forcing on supra-regional-scale^18^. For example, cave δ^18^O records from different sites across both India and eastern China show similar signatures on orbital to millennial timescales, regardless of large differences in local precipitation amount or precipitation minus evaporation (P-E) condition at each cave site^16^,^18^,^20^,^21^,^22^, suggesting that cave δ^18^O variations result mainly from changes in large-scale atmospheric circulation, rather than local precipitation amounts that vary significantly within the AM domain on various spatial-scales. This is analogous to the same annual pattern of modern precipitation δ^18^O in eastern China (Supplementary Fig. 6), where precipitation amount varies considerably over the same region.

We previously had established the supra-regional pattern of δ^18^O_p_ variability in eastern CA based on the Kesang δ^18^O record^9^. The strong covariance of δ^18^O records from Ton and Kesang caves (more than 1000 km away from each other) (Fig. 2) corroborates that they reflect changes in the supra-regional climate. Alike to the AM δ^18^O record (Fig. 2; Supplementary Fig. 7), the orbital-scale δ^18^O_p_ variation in CA inversely follows Northern Hemisphere summer insolation (NHSI) and superimposed millennial events occur as pronounced excursions that can be correlated one-to-one to Greenland stadials/interstadials (Fig. 2). This inferred pattern of climate variability in CA is well consistent with recent modeling studies^2^,^25^ with cave orbital δ^18^O variations corresponding inversely to the annual precipitation amount averaged on supra-regional scale in CA^5^ (Fig. 2C).

### Cave δ^13^C proxy

Speleothem δ^13^C and trace element variations are largely controlled by local processes at cave sites, such as changes in local hydrology, soil and vegetation dynamics, and effective infiltration^26^,^27^,^28^,^29^. Under humid conditions, the combination of a dense vegetation cover, enhanced soil microbial productivity, and high drip rates (more rapid infiltration associated with reduced degassing of CO_2_ from dripwater) leads to more negative δ^13^C values^30^,^31^,^32^. In contrast, a reduction of local effective infiltration, decrease in soil bioproductivity and reduced drip rates which allows for prolonged degassing result in higher δ^13^C values of the speleothems^29^. Although other factors could potentially affect the δ^13^C values, for example, the kinetic fractionation, variable organic carbon content and vegetation type, the observed relationship of δ^13^C with δ^18^O and trace element data, support our interpretations outlined below (see the following section as well). We suggest that the notably higher δ^13^C values of Ton records (Fig. 1) may result from the lack of vegetation in the Ton cave. Considering that the Ton samples have δ^13^C values typically near the host rock value (~2.6‰), it is conceivable that the major carbon source was the host rock. The further enhanced δ^13^C values above 2.6‰ may most likely result from the prior calcite precipitation (PCP) process in the epikarst, which is consistent with the trace element data as explained below. However, in principle kinetic effects can also potentially increase the δ^13^C values to some extent.

### Trace element proxy

**Figure 3:**
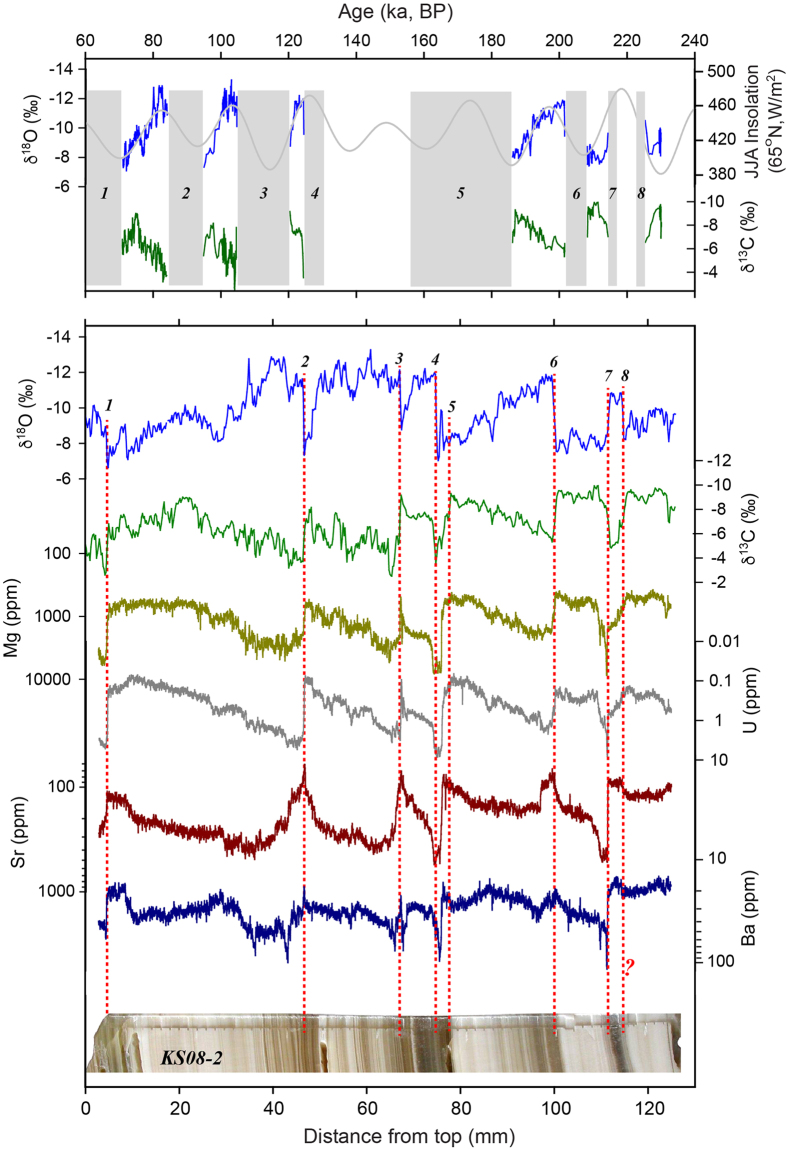
Comparison between stable isotope and trace element records of Kesang stalagmite KS08-2. Upper panel: stable isotope time series and hiatuses (1 to 8)^9^. Lower panel: stable isotope and trace element data (5-point running mean) on a distance scale. Similar to the correlations observed in other Kesang samples (Supplementary Figs 9 and 12), there is a significant correlation between δ^13^C and Mg, U, Sr and Ba records, while the δ^18^O data show distinct patterns with an overall out-of-phase/anti-phase relation to both δ^13^C and trace element records.

Ton and Kesang trace element (Mg/Ca, Sr/Ca, S/Ca, Ba/Ca and U/Ca) records generally exhibit coherent variations among themselves as well as with the corresponding δ^13^C records (Figs 1 and 3; Supplementary Figs 8–13). The replicating nature of these records obtained from different samples from the same cave (Fig. 1), provides a robust test of their reliability.

In general, speleothem Mg/Ca, Sr/Ca and Ba/Ca ratios are controlled by parent water composition and speleothem growth rate^33^,^34^. However, our Ton and Kesang trace element data do not appear to be sensitive to growth rates. The PCP has been shown to be a common process in the vadose zone of many cave systems, which considerably affects Mg/Ca, Sr/Ca and Ba/Ca ratios in stalagmites^28^,^35^,^36^,^37^,^38^. This is because, under drier conditions, reduced recharge of the karst aquifer leads to more intensive CO_2_ degassing from water into air pockets in the epikarst, as well as water entering the cave, resulting in progressive enrichment of Mg, Sr and Ba, relative to Ca in the seepage water, and subsequently in the stalagmites. In contrast, less PCP occurs during wet conditions, resulting in lower Mg, Sr and Ba concentrations relative to Ca in the seepage water and thus in the stalagmites^28^,^35^. The broadly positive correlation between the trace element data (Supplementary Figs 8–13) is supportive of this interpretation^39^.

The general positive correlation between the trace element (Mg/Ca, Sr/Ca and Ba/Ca) data and δ^13^C observed in both Kesang and Ton records further corroborates the role of the PCP^36^. Under drier conditions, enhanced PCP gives rise to higher δ^13^C values in dissolved inorganic carbon of the seepage water^36^,^40^,^41^ and, as aforementioned, prolonged CO_2_-degassing allows for ^13^C enrichment in speleothem carbonate^26^. As such, a co-variation of δ^13^C values and trace element concentrations is most likely related to the degree of amelioration of the soil, epikarst and karst aquifer overlying the cave, which in turn is linked to P-E conditions at cave site. In addition, sulfur aerosols in CA are mainly derived from gypsum sourced from the Caspian and Aral Sea regions^42^. Thus, higher sulfur contents in the Ton stalagmites may indicate increased atmospheric dust transport under drier conditions, associated with increased dust and gypsum in the source region and *vice versa*. This is supported by the observed strong covariance between S/Ca, Sr/Ca and δ^13^C in Ton records (Fig. 1; Supplementary Fig. 13).

## Discussions

While the Ton and Kesang δ^18^O records are coherent, their δ^13^C and trace element records show clear differences, except during the Holocene (Figs 1 and 2). This observation is consistent with the nature of the proxies: δ^18^O is a proxy of changes in atmospheric circulation on a supra-regional scale besides local conditions; and δ^13^C and trace element data essentially reflect local P-E conditions at cave areas that may have large spatial disparity. Although other possibilities may exist, the above interpretation is more coherent, in regard to the replications between different samples, as well as the constraints from the covariance between δ^13^C and trace elements.

Hitherto, a lack of high-resolution, precisely-dated and multi-proxy climate records has hampered efforts to reliably test the coupling relationship between the Westerly and AM climates as suggested by recent climate model simulation and data reanalysis^2^,^3^,^4^,^5^,^10^. We have previously demonstrated that the Kesang δ^18^O record, which represents the boreal spring-summer precipitation regime in eastern CA, is dominated by precession cycles that closely track NHSI inversely. The new Ton δ^18^O record from a typical boreal wintertime precipitation dominated area in the core of CA shows the same orbital-scale variability (Figs 1 and 2). Furthermore, the millennial-scale events superimposed on both records (namely CA interstadials/stadials) are also clearly coincident with Greenland/AM interstadials/stadials documented in Greenland ice cores and Chinese cave AM records (Fig. 2). In this light, the supra-regional variability is not only coherent in both regimes within Westerly CA that are dominated by winter and summer precipitation respectively, but also closely linked to AM on orbital to millennial timescales, including glacial terminations I and II, the Younger Dryas (YD) event, most of the interstadials between 8 and 25, as well as Marine Isotope Stages (MIS) 1, 3 and 5 (Fig. 2, Supplementary Fig. 7). These results strongly support the notion that the Westerly and the AM climates are closely coupled as a large system on a wide range of timescales via changes in the Westerly-Jet strength and the position relative to the Tibet Plateau, as well as in the associated atmospheric circulations^1^,^2^,^3^,^4^,^10^.

A close inverse correspondence between orbital-scale variations in CA cave δ^18^O records and NHSI can be explained by the relative increase of wintertime precipitation in CA during high NHSI periods^7^,^9^,^25^. This is because higher NHSI is accompanied by lower winter insolation, which intensifies the Mediterranean storm track and thus increases winter precipitation with typically low δ^18^O_p_ value^25^. In addition, the colder winter may further reduce the δ^18^O_p_ value via the temperature effect^9^, resulting in the large amplitude of precession variations in the δ^18^O_p_ as observed in cave records.

The registration of millennial to centennial scale events in CA δ^18^O records however, appears more complex. Whereas large-scale millennial events are recorded as pronounced heavier δ^18^O excursions (e.g., the YD and 105 ka events in the Ton records and the event between Greenland interstadials 20 and 21 in the Kesang record, Fig. 2), centennial-scale events during the Holocene, such as the Dark Age Cold Period (DACP) and Little Ice Age (LIA), display opposite (or only minor) δ^18^O excursions (Supplementary Fig. 14). The complexity may result from different glacial and interglacial boundary conditions. The Holocene centennial-scale events occurred under typical interglacial boundary conditions that may differ substantially from the YD and other aforementioned millennial-scale events occurred during glacial time periods. Indeed, in terms of effective moisture, the millennial-centennial events in AM and CA are reported to be out-of-phase in the late Holocene and in-phase in the last glacial period, respectively^9^,^43^,^44^.

Since AM changes are interconnected to the position and strength of the Westerly-Jet on various timescales^1^,^2^,^3^,^4^,^10^, the CA δ^18^O variations that are coupled with the AM changes should presumably link to the same jet variation and associated changes in atmospheric circulation and moisture source (Supplementary Fig. 6). A recent reanalysis of modern precipitation δ^18^O_p_ and ice core data in CA provided new insight into the mechanism driving the δ^18^O_p_ variability^45^. It is clear that modern δ^18^O_p_ variations on a seasonal-scale are mainly controlled by a ‘temperature effect’, a significant positive correlation between δ^18^O_p_ and temperature^9^, however, on interannual to decadal timescales, an inverse correlation between weighted average δ^18^O_p_ and temperature are observed. This is because the warm period corresponds with the more moisture with more remote source, longer trajectory and thus lighter δ^18^O_p_ value and *vice versa*^45^. Liu *et al*.^45^ concluded that those δ^18^O_p_ variations in CA are primarily controlled by changes in moisture source which are ultimately caused by northward-southward shifts of the Westerly-Jet. This mechanism might be extrapolated to explain the millennial-scale event in our cave records with cold events corresponding to heavier δ^18^O_p_ values^45^. Nevertheless, more high-resolution records and model simulations are urgently needed to further assess the δ^18^O changes in CA on millennial to centennial timescales.

**Figure 4:**
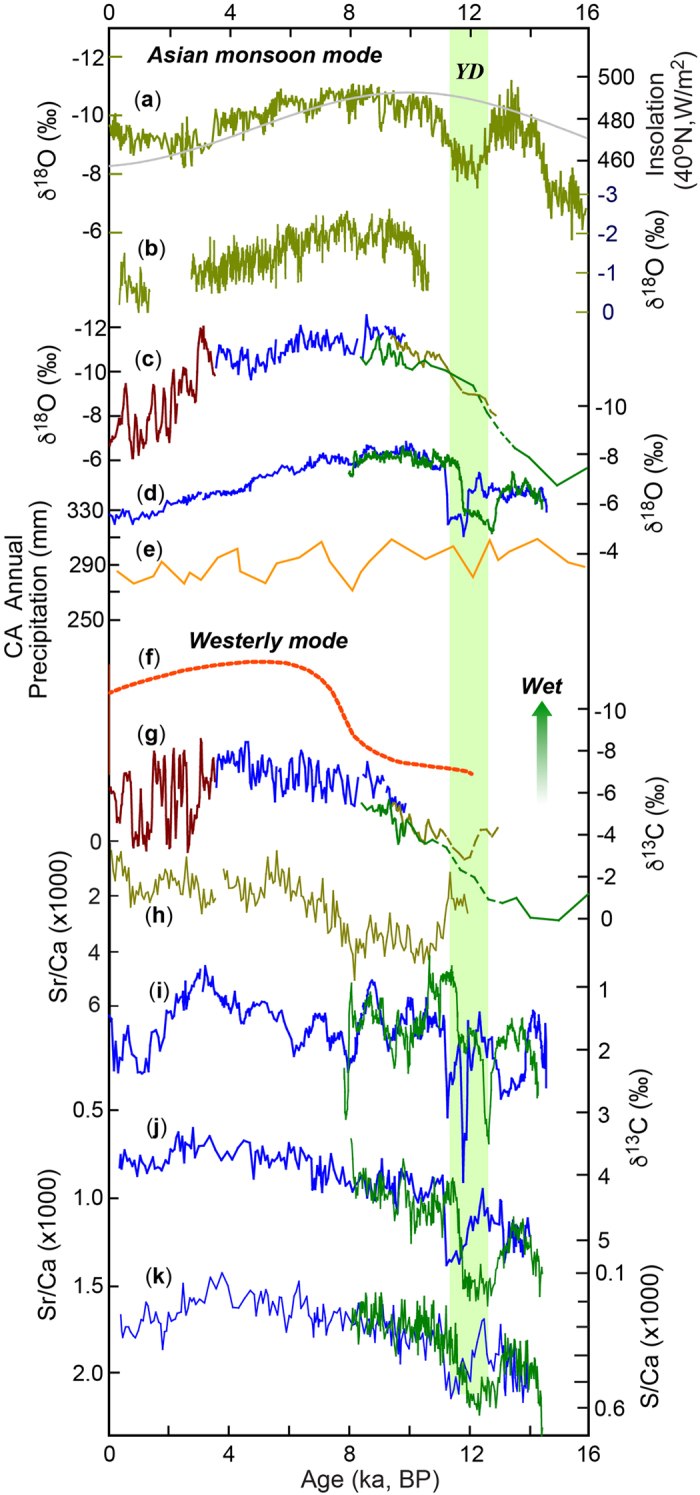
Holocene climate modes in CA. Two Holocene climate modes inferred from δ^18^O (supra-regional) and δ^13^C/trace element (cave area) records from Kesang and Ton caves, respectively. (**a**,**b**) Asian monsoon δ^18^O records (East Asian^18^ and Indian monsoon^58^, respectively). (**c**,**d**) CA δ^18^O records from Kesang^9^ and Ton (this study) caves, respectively. (**e**) Modeled annual precipitation in CA^5^. (**f**) The CA climate mode inferred mainly from lake records^6^. (**g**,**h**) Kesang δ^13^C and Sr/Ca records. (**i**–**k**) TON-1 (green) and TON-2 (blue) δ^13^C, Sr/Ca and S/Ca records, respectively. While the supra-regional hydroclimatic mode (**c**–**e**) is similar to the AM (**a**,**b**), the cave site hydrological mode (**g**–**k**) is consistent with the published lake records in the same region (**f**) with a phase lag of several ka to the supra-regional mode.

The δ^13^C and trace element records from Ton and Kesang caves exhibit coherent changes, but considerably different from their corresponding δ^18^O records (Figs 1, 2, 3). The Kesang δ^13^C record is broadly in phase with trace element records, but out-of-phase or anti-phased with its δ^18^O record on orbital to centennial timescales (Figs 1 and 3; Supplementary Figs 9–12). This observation suggests that the hydrological conditions in the Kesang area became wetter (low δ^13^C and trace element values) during low NHSI periods or cold events (such as the Greenland stadials) (corresponding to high δ^18^O values) and *vice versa*. Interestingly, the relationship on orbital timescales between Kesang δ^13^C and δ^18^O values during both the Holocene and MIS 11 is not strictly anti-phased as seen typically for other time periods over the past 500 ka. Instead, they are merely out-of-phase with δ^13^C variations lagging δ^18^O by several ka (Fig. 4; Supplementary Fig. 10). Regarding millennial to centennial events, δ^13^C and δ^18^O variations are positively correlated during the late Holocene, in contrast to the negative correlation observed throughout in other time periods (Supplementary Fig. 11). Consistently, new Kesang data in the Late Holocene also show more negative excursions in both δ^18^O and δ^13^C values during the DACP than during the Medieval Climate Anomaly (MCA) time (Supplementary Fig. 14), in line with the observations of a wetter^7^,^46^,^47^ and lower cave δ^18^O value LIA (Y.J. Cai, personal communication, 2016). However, further investigation is needed, because our new DACP and LIA records are short and incomplete. In addition, we suspect that the Holocene and possibly MIS 11 as well, are distinguished among interglacial periods by low eccentricity (and thus low precession change) at a pacing of ~400 ka eccentricity cycle, and the smaller NHSI variations would have suppressed changes in the Siberian High, an atmospheric high pressure system, which affects the Westerly-Jet position, and in turn, moisture transport at the Kesang site^9^,^48^, leading to a distinct response of the hydroclimate changes. Nevertheless, more observational and modeling studies are needed to further assess this proposition.

Ton δ^13^C, Sr/Ca and S/Ca records are broadly correlated (Supplementary Fig. 8) but, unlike the Kesang records, do not exhibit a negative correlation with δ^18^O. Instead, δ^13^C and δ^18^O display a weak positive correlation (Supplementary Fig. 13). The precession cycles that dominate the δ^18^O record are virtually absent in the δ^13^C, Sr/Ca and S/Ca records (Fig. 1). On the millennial timescale, the prominent cold events correspond with moderately drier conditions, including the YD event, the 105 ka event in the middle of MIS 5c, and numerous CA stadials recorded in the Ton δ^13^C and trace element records (Figs 1 and 2). It is evident that the hydroclimate variations in Kesang and Ton cave areas are distinct and generally respond differently to supra-regional climate changes in CA.

Notably, the Holocene variations of effective moisture (or P-E) inferred from δ^13^C and trace element data from both Kesang and Ton caves appear to lag the regional δ^18^O change, peaking at ~4 ka ago later than the δ^18^O minimum at ~10 ka ago. This P-E pattern inferred from the cave data in the Holocene appears to have a large spatial extent in CA, since a wide range of lake records in CA also display a virtually same pattern^6^ (Fig. 4). This pattern of climate change in Holocene, referred to as CA hydroclimate mode^6^, is clearly different from or out-phased with the AM δ^18^O record. This is not surprising because the former indicates local P-E condition in CA and the latter shows large-scale changes in spatially-integrated AM rainfall.

The lag of the effective moisture or P-E changes during the Holocene to the supra-regional change inferred from the δ^18^O record has been attributed to changes in the North Atlantic sea-surface temperature (SST) and high-latitude air temperature, which control moisture availability, amount and transport^3^,^6^. An alternative explanation invokes changes in P-E conditions at precession bands. Higher summer insolation during the early Holocene may have intensified summer evaporation substantially and resulted in an overall lower annual P-E value in comparison to the middle-late Holocene. This mechanism agrees with the model simulations that show a low and steady amount of annual precipitation broadly tracking NHSI in CA during the Holocene^5^ (Figs 2 and 4), and thus higher NHSI would result in a lower P-E value in the early Holocene, consistent with observations from both lake and cave data.

A closer examination of Ton and Kesang records beyond the Holocene shows that the above-mentioned CA climate mode appears to be non-stationary, for instance, the remarkable dissimilarity in comparison to the temporal-patterns in MIS 5e to 5a (Fig. 1). The coherent Ton δ^13^C and trace element records beyond the Holocene show a weak and variable response to NHSI change on orbital timescales (Fig. 1). In contrast, the Kesang δ^13^C and trace element records beyond the Holocene show a nearly anti-phased relation with the corresponding δ^18^O records (Supplementary Figs 9–11). These observations demonstrate a spatially differentiated P-E history in CA beyond the Holocene, thus suggesting a possibility of two, or potentially more, modes in CA in terms of effective moisture variations prior the Holocene. In general, the spatially heterogeneous nature of effective moisture or P-E condition is common within a large climate system on various timescales. In the AM domain, for instance, the hydroclimate variations are rather distinct or out-of-phase between southeastern and northern China on almost all timescales, although cave δ^18^O records on orbital-millennial scales and modern δ^18^O_p_ change on seasonal scales are broadly identical^16^,^18^,^22^. It would also have at least two AM modes in eastern China (e.g., southern and northern China, respectively) in terms of P-E or effective moisture changes^22^,^49^,^50^, but only one single coherent mode with regard to the variability of the integrated monsoon rainfall inferred by δ^18^O proxies^5^,^16^,^18^,^22^.

We propose that the climatic modes are essentially identical in the two climatic regimes, Westerly CA and Monsoon Asia, in regard to their large-scale atmospheric circulation changes. This is evident from a wide range of previous model/reanalysis works^2^,^3^,^4^,^5^,^10^,^25^ and now supported by this study. Our results support rather than contradict to the previous hypothesis that the Holocene mode of CA based on effective moisture changes is out-of-phase with the AM.

## Summary

Both Ton and Kesang δ^18^O changes coincide with AM variations, including the Holocene variability, suggesting a close linkage between the supra-regional climate change in CA and AM climates on orbital to millennial timescales. This observation supports the hypothesis that the Westerly and the AM are fully coupled as a large system on multiple timescales, and coordinated via changes in the position and strength of the Westerly-Jet. In contrast, as inferred from the Ton and Kesang δ^13^C and trace element records, hydroclimatic changes in each cave area may be highly complex and likely different from large-scale climate changes inferred from δ^18^O records, depending on locations in CA and possibly time periods as well. Our multi-proxy data reconcile the paradox of an apparent difference in climate modes between Westerly CA and AM climates by identifying different CA climate modes on different tempo-spatial scales.

## Methods

### ^230^Th dating

The stalagmites were cut into halves along their growth axes and polished. A total of 76 subsamples (27 for TON-1, 45 for TON-2, and 4 for KS08-6) were drilled for ^230^Th dating (Supplementary Table 1). ^230^Th dating was performed at the Isotope Laboratory of Xi’an Jaiotong University, China. We use standard chemistry procedures to separate U and Th, as described in ref. 51. The isotope dilution method with a triple-spike ^229^Th–^233^U–^236^U was employed to correct for instrumental fractionation and determine U-Th isotopic ratios and concentrations. Measurements of U-Th isotopic ratios were performed on a Multi-Collector Inductively Coupled Plasma Mass Spectrometer (Thermo-Scientific Neptune). The instrumentation, standardization and half-lives are reported in refs 13 and 52. All U-Th isotopes were measured on a MasCom multiplier behind the retarding potential quadrupole in the peak-jumping mode. We followed similar procedures of characterizing the multiplier as described in ref. 52. Uncertainties in U-Th isotopic data were calculated offline at the 2σ level, including corrections for chemistry/instrument blanks, multiplier dark noise, abundance sensitivity, tails, and contents of the same four nuclides in the spike solution^13^,^52^. Corrected ^230^Th ages assume an initial ^230^Th/^232^Th atomic ratio of 4.4 ± 2.2 × 10^−6^, the values for a material at secular equilibrium with the bulk Earth ^232^Th/^238^U weight ratio of 3.8.

### Stable isotope measurements

The stable oxygen and carbon isotopic composition of stalagmite samples was analyzed at two laboratories, the Institute of Geology, Universität Innsbruck, Austria (stalagmites TON-1 and TON-2 from Ton cave) and the Isotope Laboratory of Xi’an Jaiotong University, China (stalagmites KS08-6 from Kesang cave). Results are reported in per mil (‰), relative to the Vienna PeeDee Belemnite (VPDB) (Supplementary Table 2). The techniques used at Universität Innsbruck are described in ref. 53. Stable isotope samples were micromilled perpendicularly to the extension axes of the stalagmites at 0.05 to 0.1 mm increments and analyzed using an on-line carbonate preparation system (Gasbench II) interfaced with an isotope ratio mass spectrometer (DeltaplusXL). Stable isotopes of KS08-2 were determined on a Finnigan MAT-253 mass spectrometer with an online carbonate preparation system (Kiel-IV). Standard measurements have an analytical precision (1σ) of typically 0.08‰ for δ^18^O and 0.06‰ for δ^13^C in both laboratories.

### Trace element analysis

(1) XRF. High-resolution Sr/Ca and S/Ca data from all Kesang and Ton samples were obtained by micro-X-ray fluorescence (XRF) scanning using an ITRAX core scanner at the State Key Laboratory of Geological Processes and Mineral Resources, China University of Geosciences, China. The method is described in detail in ref. 54. Since the width of the track is about 1 cm, precise correlation with the stable isotope data is difficult. However, the overall correlation is still obvious on orbital time scale (Supplementary Figs 8–13).

(2) Laser ablation inductively coupled plasma mass spectrometry. We obtained high-resolution trace element data for sample KS08-2 using a Thermo Finnigan Element 2 Inductively Coupled Plasma Mass Spectrometer (ICPMS) coupled to a New Wave UP213 Laser Ablation system at the Max Planck Institute for Chemistry, Germany. The isotopes ^25^Mg, ^43^Ca, ^88^Sr, ^137^Ba, and ^238^U were analyzed, and ^43^Ca was used as an internal standard. We used the linescan technique with a round spot with a diameter of 55 μm and a scan speed of 10 μm per second. Two separate sections were scanned parallel to the primary transect to confirm that the observed variations are not related to sample heterogeneity. Both sections had a length of approximately 10 mm. Further details of the method and evaluation procedure are reported in refs 55 and 56.

### Statistics Analysis

The correlation coefficients between different records are obtained using the bootstrap resampling method. The sample size of each resampling is 200, and the total resampling performed is 2000. The sample size of each resampling is 200 (50 for the dataset with total size less than 500). The total resampling performance is 2000, which provides a significance level of *p* < 0.05 or < 0.01 for the expectation of correlation coefficient. The results of statistical analyses are shown in Supplementary Figs 7, and 11–13. The red curves in the figures are the probability density curves fitted by the Gaussian distribution. The correlation coefficients are labeled together with standard errors. The ranges of these correlation coefficients (shadow areas in the figures) generally do not cross the origin point, further demonstrating their statistical significances. We also calculate the Pearson correlation coefficients for the paired datasets. All of them are close to the expectations labeled in the figures with statistical significances at *p* < 0.01 or *p* < 0.05 level as indicated in each figure caption.

## Additional Information

**How to cite this article**: Cheng, H. *et al*. Climate variations of Central Asia on orbital to millennial timescales. *Sci. Rep.* **6**, 36975; doi: 10.1038/srep36975 (2016).

**Publisher’s note:** Springer Nature remains neutral with regard to jurisdictional claims in published maps and institutional affiliations.

## Supplementary Material

Supplementary Information
