# Author Correction: Climate variations of Central Asia on orbital to millennial timescales

**DOI:** 10.1038/s41598-021-92496-2

**Published:** 2021-06-21

**Authors:** Hai Cheng, Christoph Spötl, Sebastian F. M. Breitenbach, Ashish Sinha, Jasper A. Wassenburg, Klaus Peter Jochum, Denis Scholz, Xianglei Li, Liang Yi, Youbing Peng, Yanbin Lv, Pingzhong Zhang, Antonina Votintseva, Vadim Loginov, Youfeng Ning, Gayatri Kathayat, R. Lawrence Edwards

**Affiliations:** 1 Institute of Global Environmental Change, Xi’an Jiaotong University, Xi’an, China; 2 Department of Earth Sciences, University of Minnesota, Minneapolis, USA; 3 Institute of Geology, Universität Innsbruck, Innsbruck, Austria; 4 Sediment- & Isotope Geology, Institute for Geology, Mineralogy & Geophysics, Ruhr-Universität, Bochum, Germany; 5 Godwin Laboratory, Department of Earth Science, University of Cambridge, Cambridge, UK; 6 Department of Earth Sciences, California State University Dominguez Hills, Carson, USA; 7 Institute for Geosciences, Johannes Gutenberg-Universität Mainz, Mainz, Germany; 8 Climate Geochemistry Department, Max Planck Institute for Chemistry, Mainz, Germany; 9 Sanya Institute of Deep Sea Science and Engineering, CAS, Sanya, China; 10 School of Geological Sciences and Mineral Resources, Lanzhou University, Lanzhou, China; 11 Nuffield Department of Clinical Medicine, University of Oxford, Oxford, UK; 12 Ekaterinburg Speleological Club (ESC), Ekaterinburg, Russia

Correction to: *Scientific Reports* https://doi.org/10.1038/srep36975, published online 11 November 2016

The Supplementary Information published with this Article contains errors where four Supplementary data tables are omitted from the final file. This data is now available in the NOAA database, and can be accessed using the link: https://www.ncdc.noaa.gov/paleo-search/study/21350.

