# Supplementary material for: Climate variations of Central Asia on orbital to millennial timescales
Source: Sci Rep. 2016 Nov 11;5:36975. doi: 10.1038/srep36975 (PMC5105073; doi:10.1038/srep36975)
Supplement: Supplementary Information [file srep36975-s1.pdf]

Climate variations of Central Asia on orbital to millennial timescales

Hai Cheng, Christoph Spötl, Sebastian F. M. Breitenbach, Ashish Sinha, Jasper. A. Wassenburg, Klaus Peter Jochum, Denis Scholz, Xianglei Li, Liang Yi, Youbing Peng, Yanbin Lv, Pingzhong Zhang, Antonina Votintseva, Vadim Loginov, Youfeng Ning, Gayatri Kathayat, R. Lawrence Edwards

Supplementary Information

1. Supplementary Figures

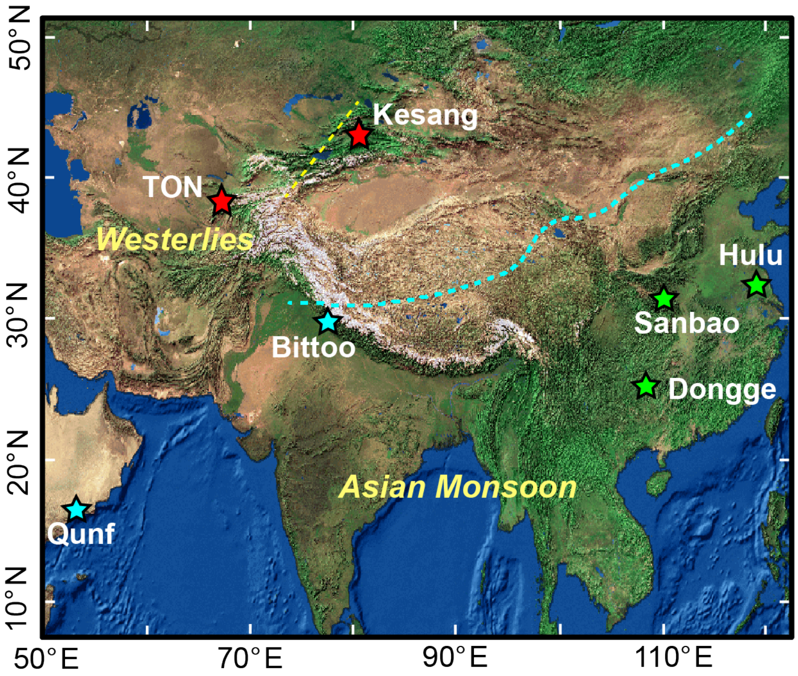

Supplementary Figure 1 | Map of cave locations. Red stars show the Central Asia caves in the Westerlies region: Ton (38°24'N, 67°14'E) and Kesang (42°52'N, 81°45'E). Green and blue stars show cave sites in Asian monsoon regions. Green stars show caves in the East Asian monsoon region: Hulu (32°30'N, 119°10'E), Sanbao (31°40'N, 110°26'E), Dongge (25°17'N, 108°5'E); blue stars show caves in the Indian monsoon region: Bittoo (30°47'N, 77°47'E) and Qunf (17°10'N, 54°18'E) caves. The light blue dashed line depicts the approximate modern fringe of the Asian summer monsoon. The light green dashed line marks the approximate boundary between winter (west) and summer (east) precipitation regimes in CA. The map is created by using the ArcGIS software (ArcGIS, [10], http://www.esrichina.com.cn/) and the world map background data are from the Environmental Systems Research Institute (ESRI).

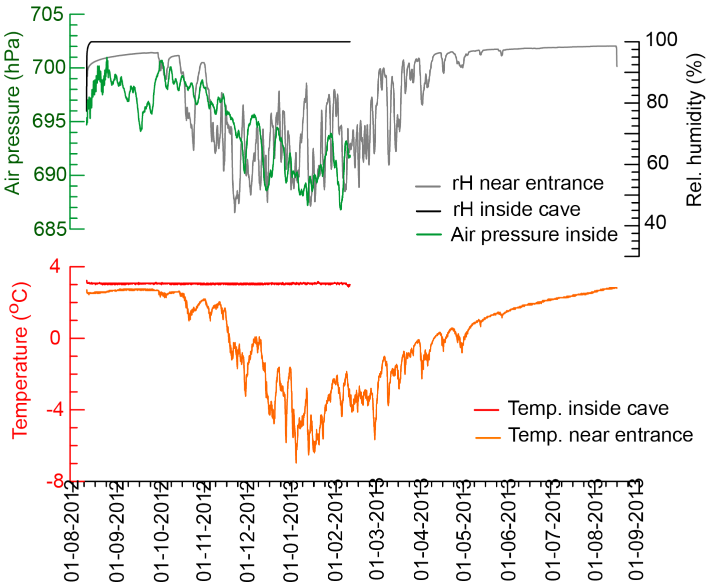

Supplementary Figure 2 | Monitoring results from Ton cave. The temperature is nearly constant (~3.1°C), and relative humidity is virtually 100% at the speleothem sampling site. In contrast, both temperature and humidity vary seasonally at the cave entrance.

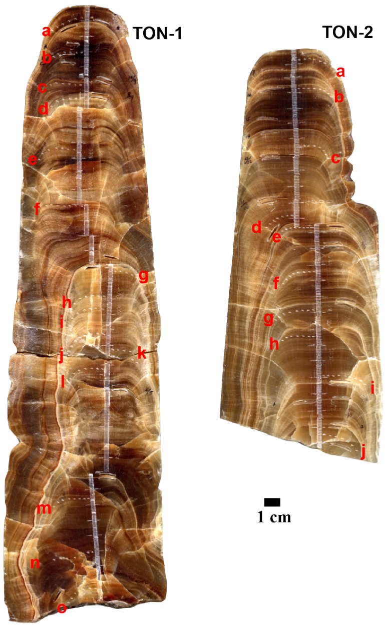

Supplementary Figure 3 | Images of stalagmites TON-1 and TON-2 from Ton cave, Uzbekistan, Central Asia. TON-1 is ~382 mm high with a diameter of ~120 mm, and TON-2 is ~246 mm high with a diameter of ~95 mm. Stable isotope subsampling tracks run along the growth axes. Letters depict the stable isotope tracks (a to o for TON-1 and a to j for TON-2) for Hendy tests[14].

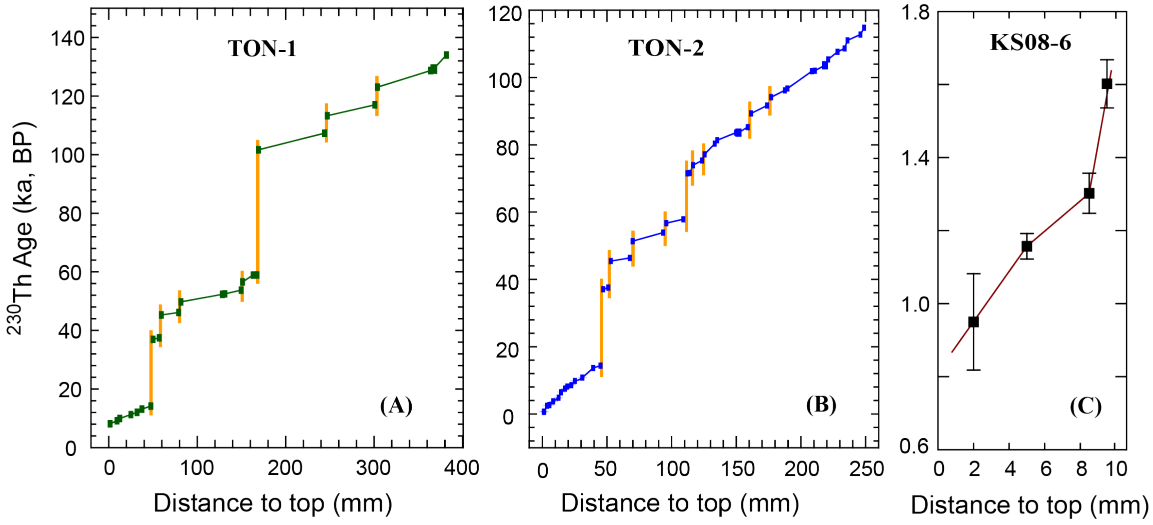

Supplementary Figure 4 | Age models of Ton and Kesang stalagmites. (**A**) and (**B**) are TON-1 and TON-2 age models, respectively. The ^{230}Th dating error is smaller than the size of the plot symbols. Vertical bars indicate hiatuses. Simple linear interpolation between ^{230}Th dates was used to establish age models. (**C**) KS08-6 age model. KS08-6 is ~10 mm high with a diameter of ~38mm.

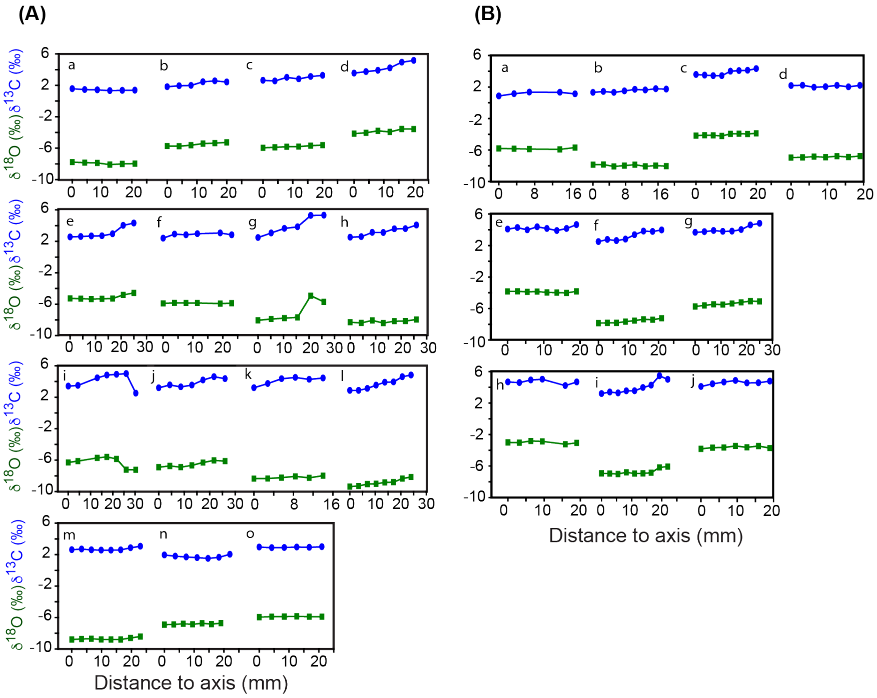

Supplementary Figure 5 | Results of Hendy Tests. (**A**) and (**B**) Hendy Test[14] on samples TON-1 and TON-2, respectively. Hendy Test positions are shown in Supplementary Figure 3. Generally, $\delta^{18}O$ and $\delta^{13}C$ values along the same layer are similar within 10 mm. However, an increase in both $\delta^{18}O$ and $\delta^{13}C$ values is isotopic fractionation is obvious towards the flanks for some tests.

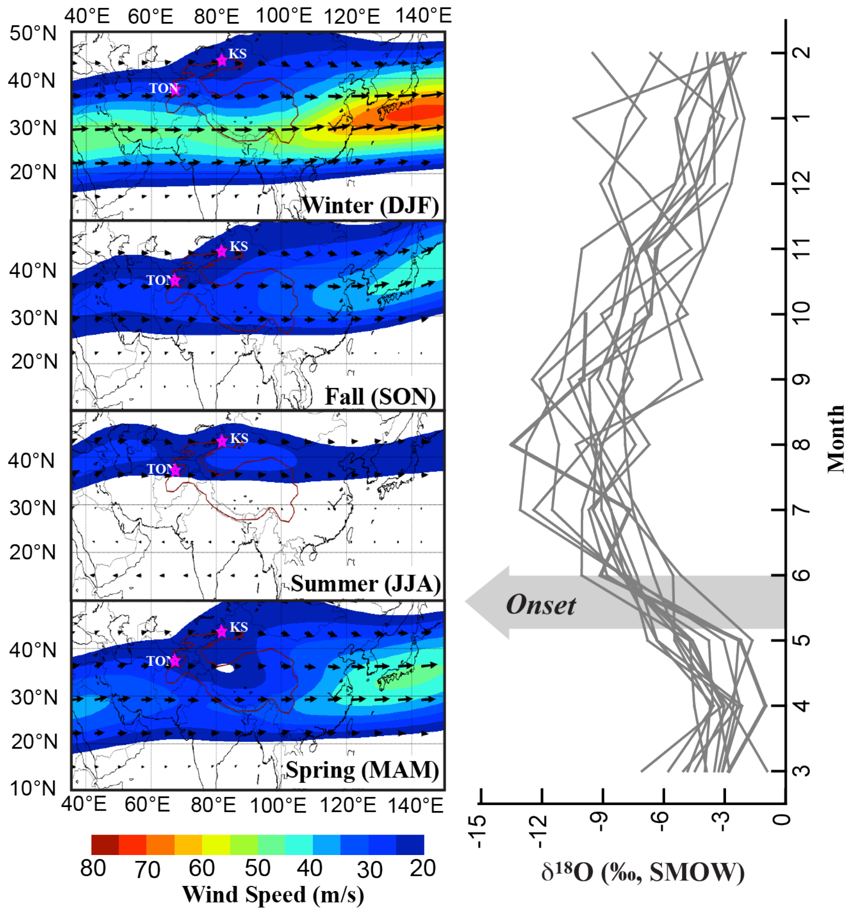

Supplementary Figure 6 | Covariance between the seasonal Westerly Jet and AM precipitation $\delta^{18}O$ values. Left panel: the seasonal change of the Westerly-Jet position and strength at 250 hPa. Right panel: monthly precipitation $\delta^{18}O$ values in the East Asian monsoon region. This co-variation on seasonal-scale is expected to occur on orbital to millennial timescales as well[2], which in turn correlates with the intensity of the Asian monsoon[16,18]. Stars show Ton and Kesang (KS) caves. Weighted monthly $\delta^{18}O$ data in the East Asian monsoon region are from IAEA/WMO (http://isohis.iaea.org). Four seasonal position/strength of the Westerly-Jet are constructed from the data of ERA-interim monthly means (http://rda.ucar.edu/datasets/ds627.1/) using the NCAR Command Language (Version 6.3.0) [Software] (2015), Boulder, Colorado: UCAR/NCAR/CISL/TDD (at http://dx.doi.org/10.5065/D6WD3XH5). The base map is from mapongly_2.ncl (http://www.ncl.ucar.edu/Applications/maponly.shtml) and also generated by the NCAR Command Language (Version 6.3.0) [Software] (2015). All the data are from the Research Data Archive at the National Center for Atmospheric Research.

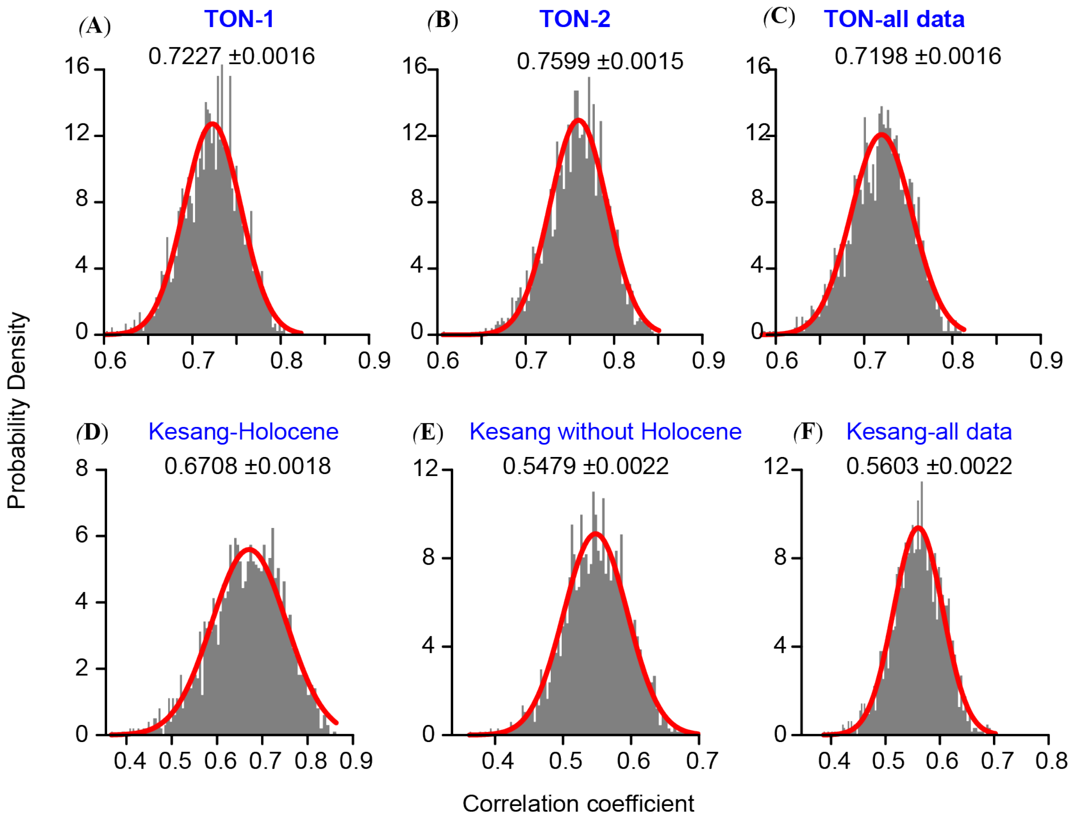

Supplementary Figure 7 | Histograms of correlation coefficients between AM and Central Asia records. (**A**) to (**C**) Histograms of correlation coefficients between the AM[18] and Ton-1, Ton-2 and both Ton-1 and -2 $\delta^{18}O$ records, respectively. (**D**) to (**E**) Histograms of correlation coefficients between the AM[18] and Kesang Holocene $\delta^{18}O$, all $\delta^{18}O$ records excluding the Holocene, and all $\delta^{18}O$ records, respectively. The AM shows significant correlations with all different CA $\delta^{18}O$ records as labeled at $p<0.01$ level.

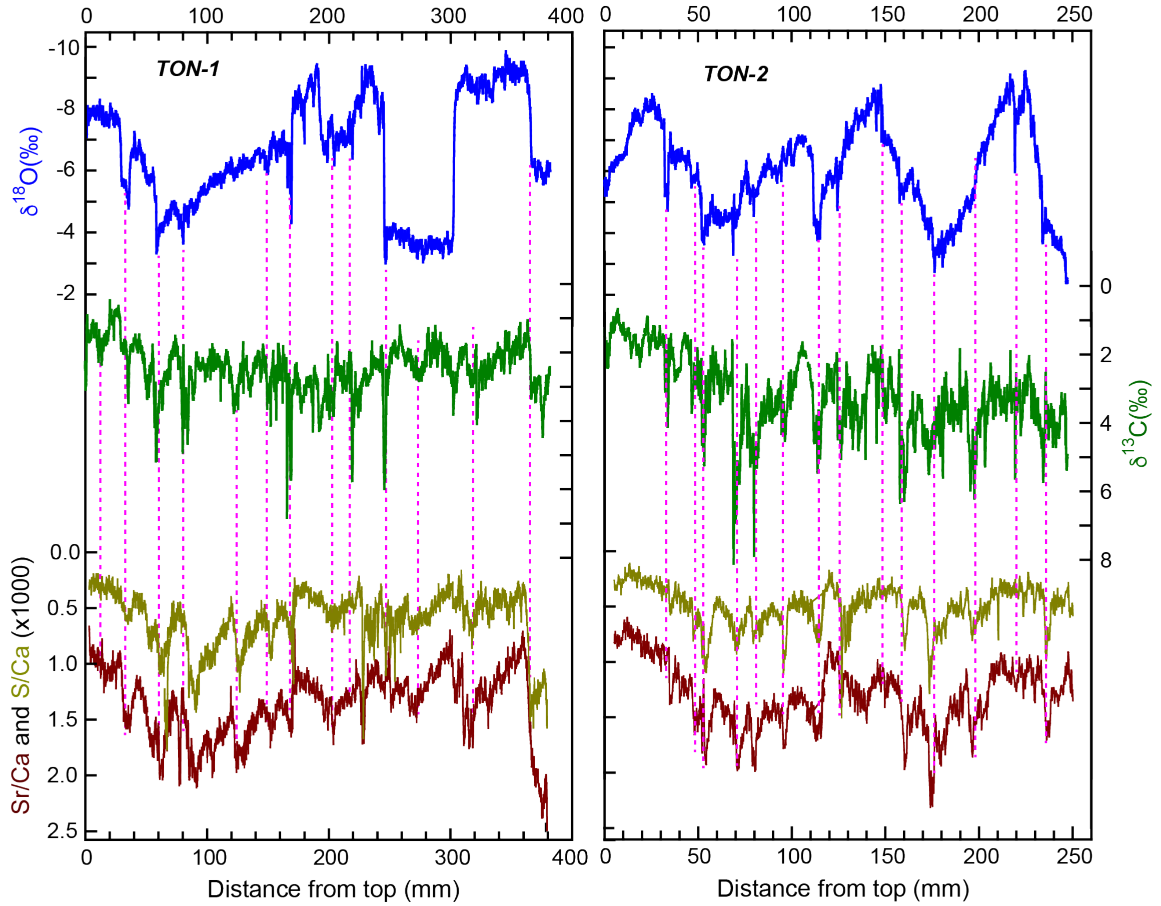

Supplementary Figure 8 | Comparison between Ton cave stable isotope and trace element records. There is a significant coherence between $\delta^{13}C$, Sr/Ca and S/Ca records for both TON-1 and TON2 samples, while the $\delta^{18}O$ data show dissimilar patterns. Sr/Ca and S/Ca are obtained by the micro-X-ray fluorescence scan.

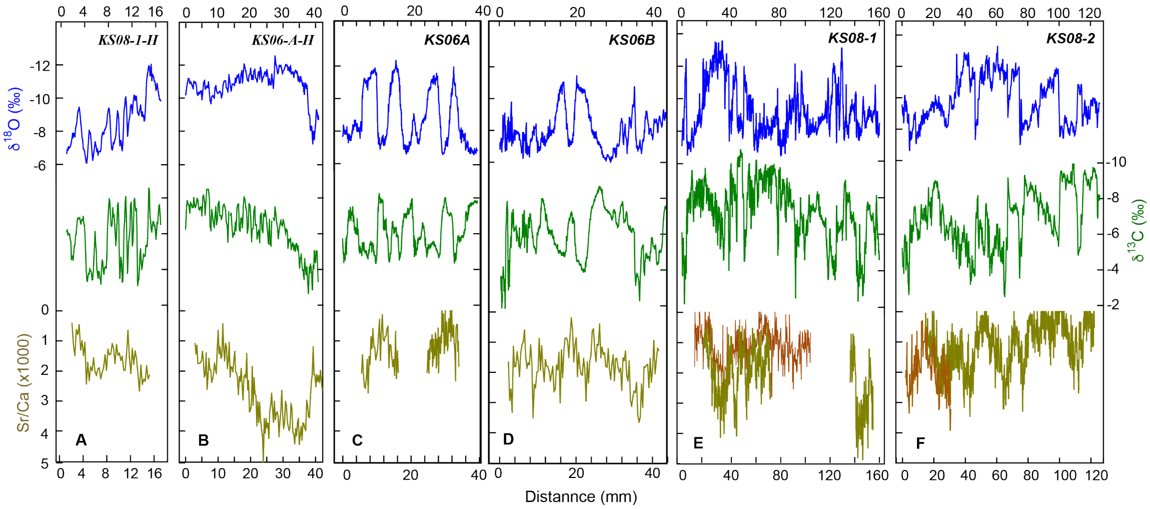

Supplementary Figure 9 | Comparison between Kesang cave stable isotope and trace element records. There is a significant similarity among $\delta^{13}C$ and Sr/Ca records for the six samples (**A** to **F**), while the $\delta^{18}O$ data show distinct patterns with an out-of-phase relation to the $\delta^{13}C$ and Sr/Ca records. Sr/Ca data are obtained by the micro-X-ray fluorescence scan. Two scan results of Sr/Ca data in (**E**) and (**F**) (in different colors) show replications.

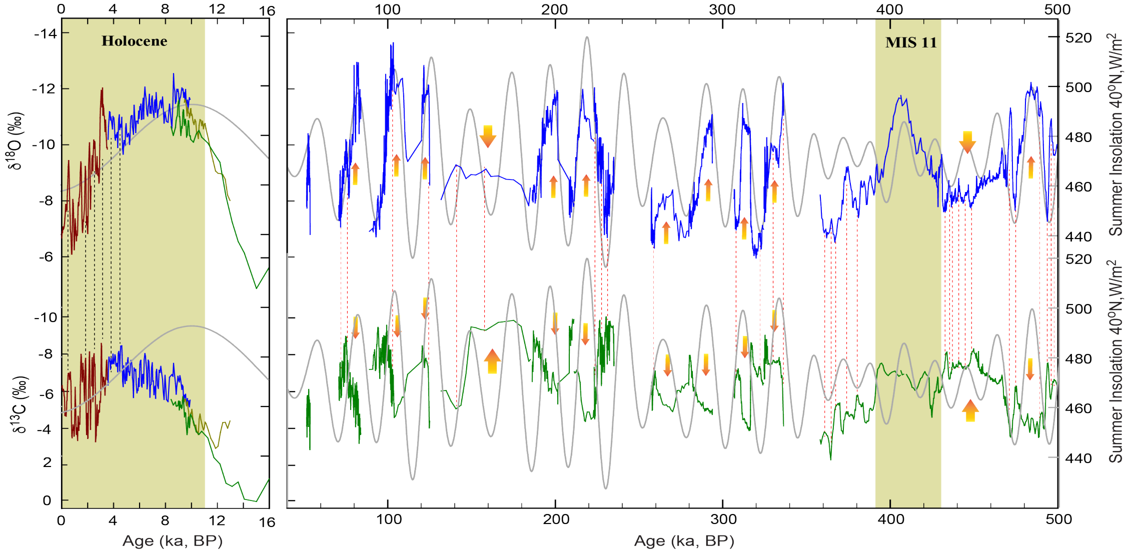

Supplementary Figure 10 | Comparison between Kesang $\delta^{18}O$ and $\delta^{13}C$ records. Similar to Asian monsoon records, the Kesang $\delta^{18}O$ variation tracks NHSI[9]. In contrast, the Kesang $\delta^{13}C$ record lags insolation and $\delta^{18}O$ variations by several ka or is nearly anti-phased (depicted by arrows) with the corresponding $\delta^{18}O$ record. For instance, the Holocene $\delta^{13}C$ record lags behind the $\delta^{18}O$ record by ~5 ka. However, the relationship appears to be non-stationary on millennial-centennial scales: the $\delta^{18}O$ and $\delta^{13}C$ variations are generally anti-correlated over most of the past 500 ka (depicted by red dashed lines), but nearly positively correlated during the late Holocene (depicted by black dashed lines) and possibly the late MIS 11.

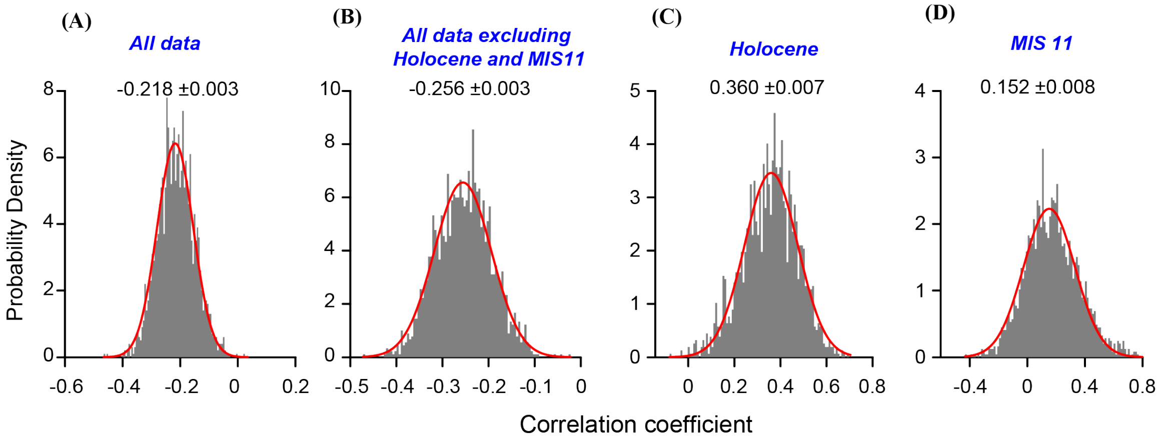

Supplementary Figure 11 | Histograms of correlation coefficients between Kesang δ^{18}O and δ^{13}C records. δ^{18}O and δ^{13}C data are from *ref.* 9. (**A**) Correlation of all the Kesang data. (**B**) Correlation of all the Kesang data excluding Holocene and MIS 11 data. (**C**) and (**D**) Correlations of Holocene and MIS 11 data, respectively. The Kesang δ^{18}O and δ^{13}C show an overall negative correlation, whereas Holocene and MIS 11 data present a slightly positive correlation (also see Supplementary Fig. 10). The calculated Pearson correlation coefficients for the paired datasets are close to the expectations labeled in each figure, and **A** to **C** are significant at $p<0.01$ level and **D** at $p<0.05$.

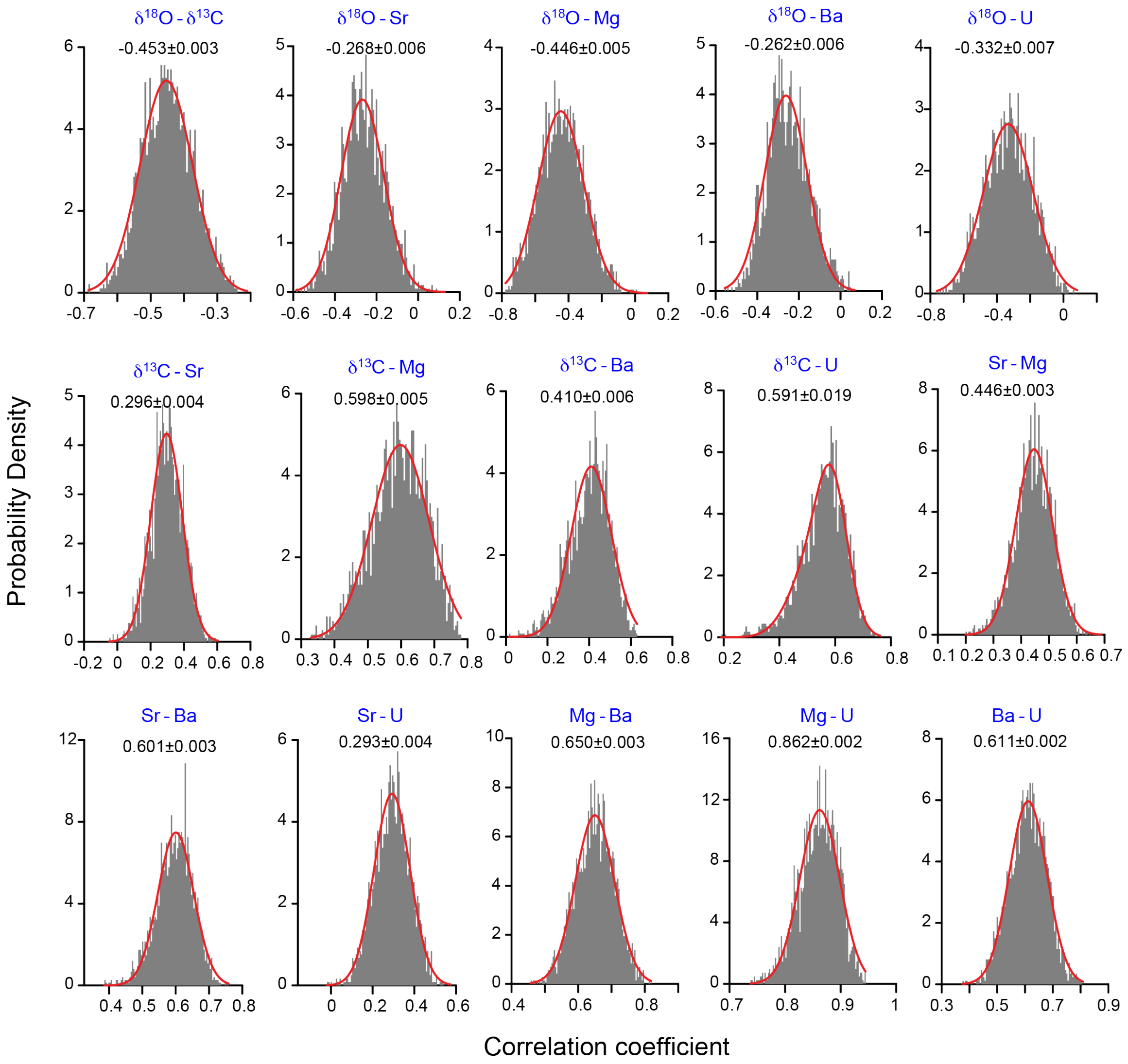

Supplementary Figure 12 | Histograms of correlation coefficients between δ^{18}O, δ^{13}C and trace elements (Mg, Sr, Ba and U) of Kesang stalagmite KS08-2. δ^{18}O and δ^{13}C data are from *ref.* 9, and trace element data from this study. The correlation coefficients are calculated after linear interpolation of the speleothem trace element data onto a common distance scale with the δ^{18}O and δ^{13}C data. Broadly, the δ^{18}O data show negative correlations with the δ^{13}C and trace element data, while δ^{13}C and trace element data show positive correlations. The calculated Pearson correlation coefficients for the paired datasets are close to the expectations labeled in each figure with significances at $p<0.01$ level.

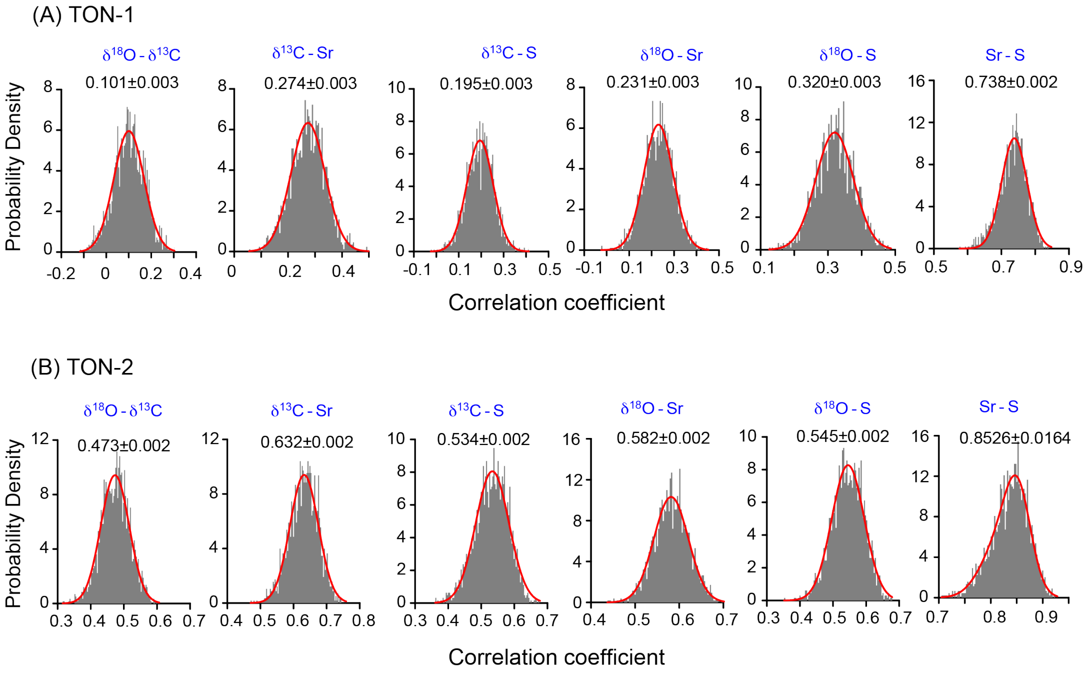

Supplementary Figure 13 | Histograms of correlation coefficients for Ton cave data. (**A**) and (**B**) Histograms of correlation coefficients between $\delta^{18}O$, $\delta^{13}C$ and trace elements (Sr and S) for TON-1 and TON-2, respectively. The two sets of data show positive correlations between $\delta^{18}O$, $\delta^{13}C$ and trace elements, which is different from the Kesang data (Supplementary Figs. 11, 13). The calculated Pearson correlation coefficients for the paired datasets are close to the expectations labeled in the figure with significances at $p<0.01$ level.

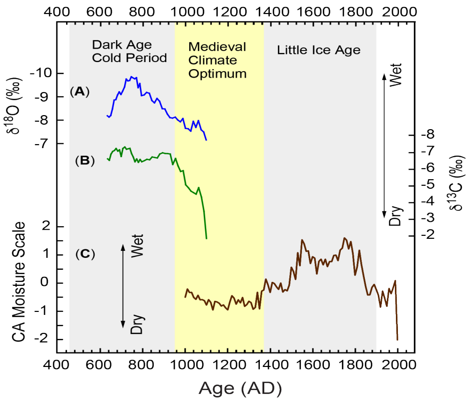

Supplementary Figure 14 | Comparison between Kesang $\delta^{18}O/\delta^{13}C$ records and the synthetic CA moisture record over the last 1500 years. (**A**) and (**B**) Kesang stalagmite KS08-6 $\delta^{18}O$ and $\delta^{13}C$ time series, respectively. (**C**) Synthetic CA moisture record[7]. Kesang $\delta^{18}O$ and $\delta^{13}C$ values during the Dark Age Cold Period are more negative (wetter) than during the Medieval Climate Anomaly consistent with the CA moisture record. The CA moisture record demonstrates a wetter Little Ice Age. However, further investigation is needed, because the DACP and LIA record is short and incomplete.

2. Supplementary Tables S1 – S4

These excel files contain all the new ^{230}Th dating results and δ^{18}O, δ^{13}C and trace element data obtained for this study (*also ready to upload to NOAA website*).
